# Crystal structure of adenosine A_2A_ receptor in complex with clinical candidate Etrumadenant reveals unprecedented antagonist interaction

**DOI:** 10.1038/s42004-023-00894-6

**Published:** 2023-06-01

**Authors:** Tobias Claff, Jonathan G. Schlegel, Jan H. Voss, Victoria J. Vaaßen, Renato H. Weiße, Robert K. Y. Cheng, Sandra Markovic-Mueller, Denis Bucher, Norbert Sträter, Christa E. Müller

**Affiliations:** 1 PharmaCenter Bonn & Pharmaceutical Institute, Department of Pharmaceutical & Medicinal Chemistry, University of Bonn, An der Immenburg 4, 53113 Bonn, Germany; 2 Institute of Bioanalytical Chemistry, Center for Biotechnology and Biomedicine, University of Leipzig, Deutscher Platz 5, 04103 Leipzig, Germany; 3 leadXpro AG, PARK InnovAARE, 5234 Villigen, Switzerland

**Keywords:** X-ray crystallography, Medicinal chemistry, G protein-coupled receptors, Mechanism of action

## Abstract

The G_s_ protein-coupled adenosine A_2A_ receptor (A_2A_AR) represents an emerging drug target for cancer immunotherapy. The clinical candidate Etrumadenant was developed as an A_2A_AR antagonist with ancillary blockade of the A_2B_AR subtype. It constitutes a unique chemotype featuring a poly-substituted 2-amino-4-phenyl-6-triazolylpyrimidine core structure. Herein, we report two crystal structures of the A_2A_AR in complex with Etrumadenant, obtained with differently thermostabilized A_2A_AR constructs. This led to the discovery of an unprecedented interaction, a hydrogen bond of T88^3.36^ with the cyano group of Etrumadenant. T88^3.36^ is mutated in most A_2A_AR constructs used for crystallization, which has prevented the discovery of its interactions. In-vitro characterization of Etrumadenant indicated low selectivity versus the A_1_AR subtype, which can be rationalized by the structural data. These results will facilitate the future design of AR antagonists with desired selectivity. Moreover, they highlight the advantages of the employed A_2A_AR crystallization construct that is devoid of ligand binding site mutations.

## Abstract (web summary)

Etrumadenant has been developed as a dual-acting adenosine A_2A_/A_2B_ receptor antagonist, and is now in clinical trials for the treatment of cancer, however, the exact drug–receptor binding mode is unknown. Here, the authors determine the high-resolution co-crystal structure of Etrumadenant with a thermostabilized A_2A_AR construct and reveal the interaction of its cyano group with T88^3.36^.

## Introduction

G protein-coupled receptors (GPCRs) activated by the nucleoside adenosine are widely distributed and play important roles in transcellular signaling^1,2^. Four subtypes of adenosine receptors (ARs) exist, the preferentially G_i_ protein-coupled A_1_- and A_3_ARs, and the G_s_-coupled A_2A_- and A_2B_ARs^3^. Coupling to additional G proteins has been described, e.g., to G_q_ proteins^4–6^. Adenosine acts as a “stop signal” resulting in strong anti-inflammatory and immunosuppressive effects, mediated by the A_2A_- and A_2B_AR subtypes^7^. Blockade of the A_2A_AR is beneficial for several pathological conditions, in which adenosine-A_2A_AR signaling is increased^8,9^. For example, the A_2A_AR antagonist Istradefylline has been approved in Japan and the USA for the treatment of Parkinson’s Disease^10^. Preclinical studies suggest major effects of A_2A_AR antagonists against Alzheimer’s Disease^11,12^. The A_2A_AR, and later on also the related A_2B_AR, both of which are expressed by immune cells and may be upregulated in cancer cells, have recently emerged as drug targets for the immunotherapy of cancer, constituting purinergic immune checkpoints^13^.

Etrumadenant (3-[2-amino-6-[1-[[6-(2-hydroxypropan-2-yl)pyridin-2-yl]methyl]-4-yl]pyrimidin-4-yl]-2-methylbenzonitrile, also known as AB928) was developed as one of the first dual-acting adenosine A_2A_/A_2B_ receptor antagonists^14^. It constitutes a unique chemotype featuring a poly-substituted 2-amino-4-phenyl-6-triazolylpyrimidine core structure. The drug has entered clinical development and has been evaluated in several clinical phase I and phase II trials for the treatment of cancer^15^. Despite its advanced stage in drug development, the characterization of Etrumadenant is limited, and the exact drug–receptor binding mode is unknown.

Here, we determined the high-resolution crystal structure of Etrumadenant in complex with a thermostabilized A_2A_AR construct comprising only two point mutations that do not interfere with ligand binding. The structure reveals unique binding pocket interactions of Etrumadenant including an interaction of its cyano group with T88^3.36^; to the best of our knowledge, this type of interaction has not been previously observed. For comparison, we also determined the high-resolution crystal structure of Etrumadenant in complex with a widely used A_2A_AR construct that contains a T88^3.36^A mutation in the binding pocket (A_2A_-StaR2-bRIL-A277S). The structural findings were complemented with an in-vitro pharmacological characterization of Etrumadenant at all AR subtypes. The compound was found to display high affinity in the low nanomolar range for A_1_-, A_2A_-, and A_2B_ARs, and it potently blocked G protein activation by these subtypes. Structural data provided an explanation for the compound’s lack of selectivity.

## Results and discussion

### Exploring the A_2A_AR binding pocket of Etrumadenant using optimized crystallization constructs

**Fig. 1:**
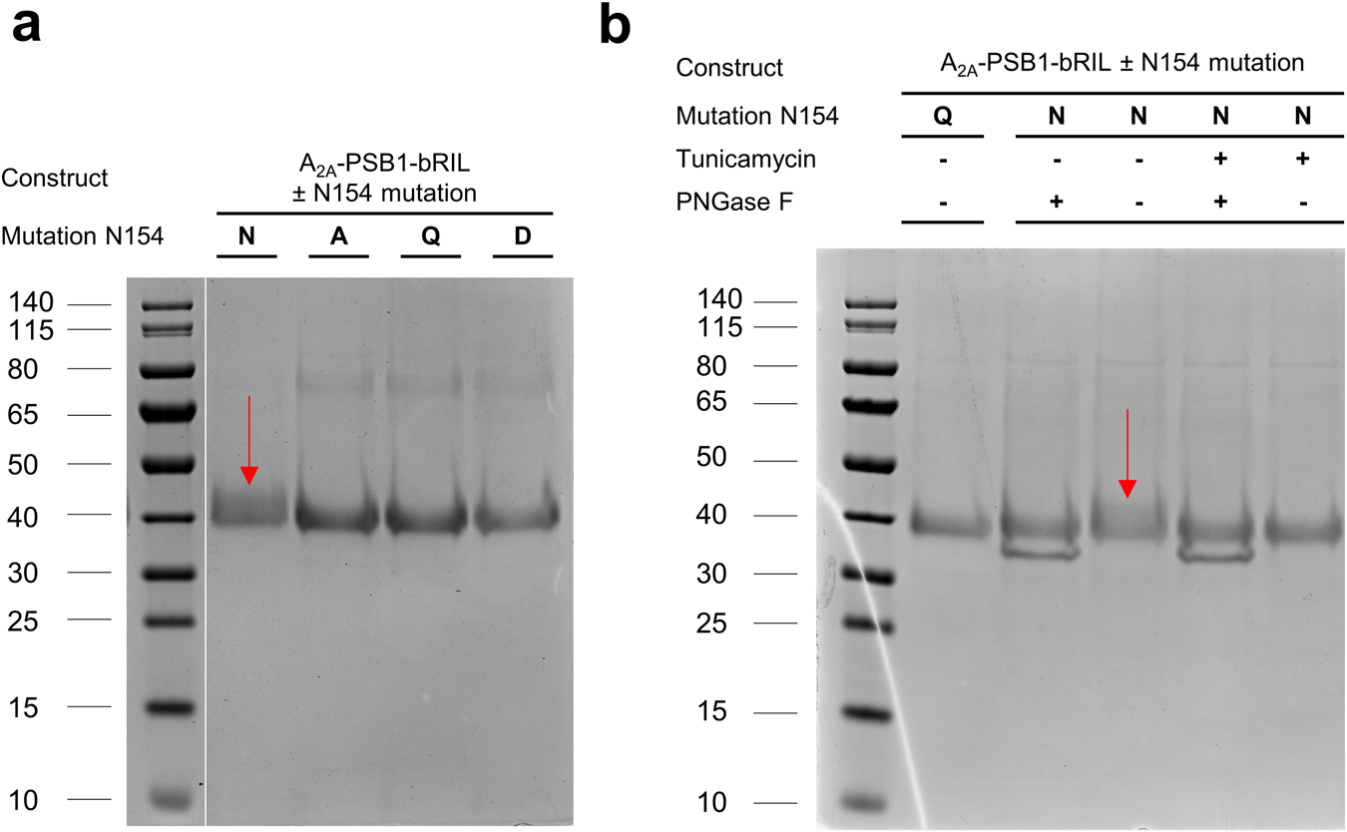
SDS-PAGE analysis of the A_2A_AR glycosylation state. **a** SDS-PAGE of A_2A_-PSB1-bRIL compared to different N154 mutations in the same protein background to remove N-linked glycosylation. The red arrow points to the characteristic glycosylation smear. A_2A_-PSB1-bRIL plus N154A corresponds to the crystallization construct A_2A_-PSB2-bRIL. The protein marker originates from the same SDS-PAGE gel. **b** The effect of Tunicamycin and PNGase F on the SDS-PAGE mobility of A_2A_-PSB1-bRIL, compared to N154Q in A_2A_-PSB1-bRIL. Equal protein amounts were loaded onto the gel. The addition of Tunicamycin during A_2A_-PSB1-bRIL expression or PNGase F treatment of the purified protein resulted in the removal of the characteristic glycosylation smear. The band for PNGase F (≈36 kDa) is visible directly below the A_2A_AR band (observed molecular weight ≈40 kDa, theoretical molecular weight ≈49 kDa). See Supplementary Fig. 1 for uncropped SDS-PAGE images.

We previously developed an optimized A_2A_AR crystallization construct designated A_2A_-PSB1-bRIL, that contains a single point mutation (S91^3.39^K) inside the allosteric sodium binding pocket to stabilize the inactive conformation which significantly enhanced protein thermostability^16^. For the co-crystallization of Etrumadenant, we used the same modification but inserted an additional point mutation (N154^ECL2^A) to remove a putative glycosylation site on extracellular loop (ECL) 2 of the receptor. This construct is designated A_2A_-PSB2-bRIL (PSB: Pharmaceutical Sciences Bonn, bRIL refers to thermostabilized apocytochrome b_562_RIL^17^). Mutation of the asparagine in position 154 to either alanine or glutamine had previously been utilized to eliminate post-translational N-linked glycosylation of the A_2A_AR, as protein glycosylation is expected to inhibit crystal growth due to microheterogeneity^18–20^. Evidence of N-linked glycosylation is missing, and N154^ECL2^ is not surface-exposed in available A_2A_AR crystal structures^21^, indicating that the non-glycosylated form of the A_2A_AR crystallizes predominantly. Here, we additionally employed sodium dodecyl sulfate polyacrylamide gel electrophoresis (SDS-PAGE) to demonstrate that A_2A_-PSB1-bRIL (bearing the wild type (wt) N154^ECL2^) is still partially glycosylated, whereas A_2A_-PSB2-bRIL (bearing an N154^ECL2^A mutation) had lost N-linked glycosylation (Fig. 1). Glycosylated proteins typically migrate more slowly in SDS-PAGE and generate higher molecular weight smearing^22^. Despite the fact that only a single glycosylation site is present, smearing could be observed for A_2A_-PSB1-bRIL, whereas a sharper band was detected for the N154^ECL2^A mutant (A_2A_-PSB2-bRIL) as well as for N154^ECL2^Q and N154^ECL2^D mutants, indicating the loss of glycosylation (Fig. 1). Alternatively, the glycosyl residues of A_2A_-PSB1-bRIL could be cleaved off by the enzyme peptide-N-glycosidase F (PNGase F)^23^ when added to the purified protein. Glycosylation could also be prevented during receptor expression by addition of the glycosylation inhibitor Tunicamycin^24^.

**Fig. 2:**
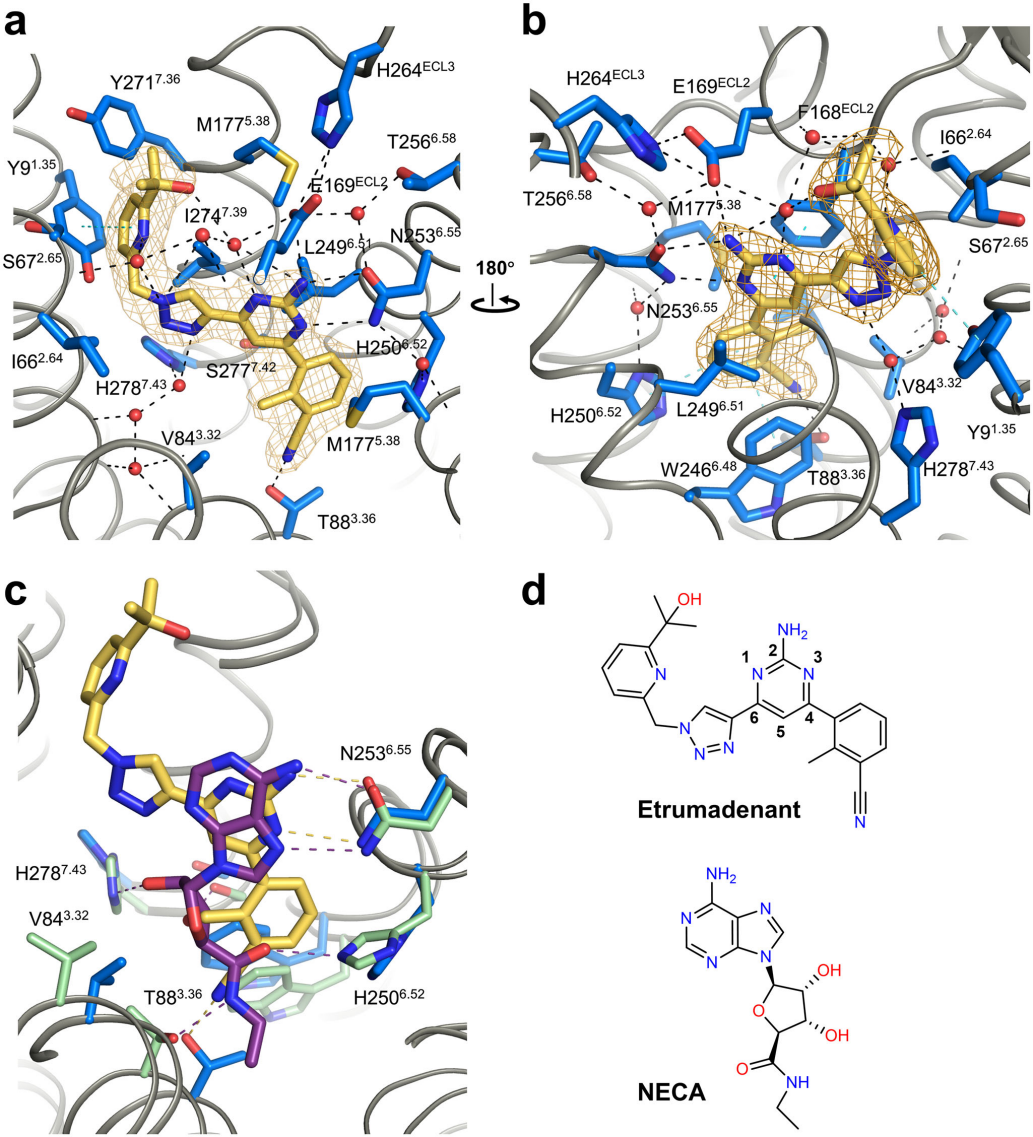
The A_2A_AR binding pocket of Etrumadenant. **a** Binding pocket of Etrumadenant with residues L167 and F168 clipped for enhanced visualization. The 2F_o_–F_c_ electron density for Etrumadenant is shown in orange mesh (contoured at 1.0 σ). **b** Binding pocket of Etrumadenant rotated by 180° compared to (**a**) with parts of the ECL2 and residues A265 to M270 clipped. The 2F_o_–F_c_ electron density for Etrumadenant is shown in orange mesh (contoured at 1.0 σ). Black dashed lines represent hydrogen bonds whereas cyan-colored dashed lines show π-π interactions. **c** Structural alignment of the A_2A_-PSB2-bRIL-Etrumadenant (blue/yellow) binding pocket with that of NECA (PDB: 2YDV, represented in green/purple). Hydrogen bonds are shown in yellow and purple, respectively. **d** Chemical structures of Etrumadenant and NECA.

**Fig. 3:**
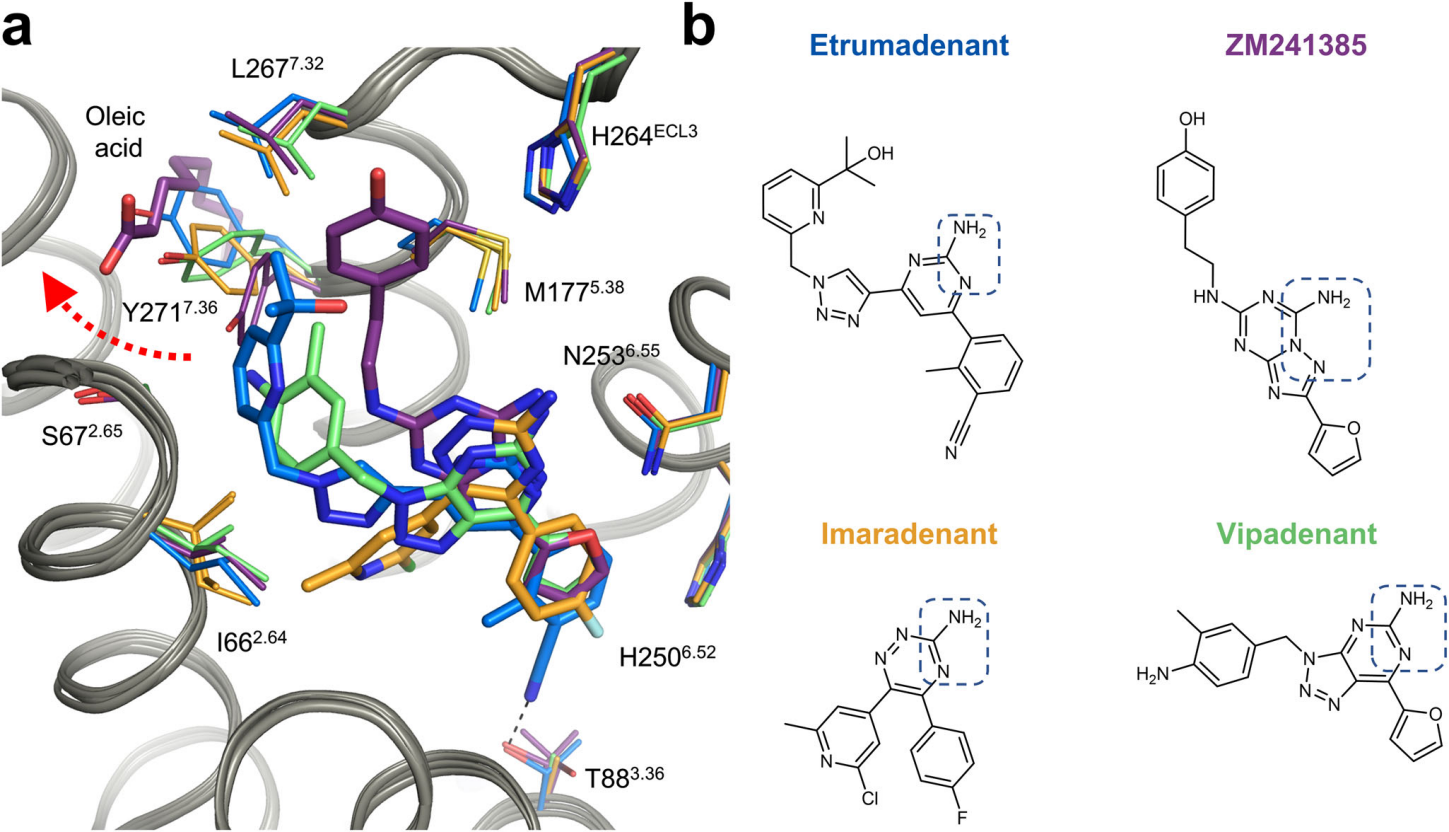
Comparison of the Etrumadenant binding pocket with that of selected A_2A_AR antagonists. **a** The binding pose of Etrumadenant (blue) is compared to the binding pockets of ZM241385 (purple, PDB ID 4EIY^21^), Vipadenant (green, PDB ID 5OLH^27^) and Imaradenant (orange, PDB ID 6GT3^26^). The red-colored dashed arrow represents the conformational movement of Y271^7.36^ in the A_2A_-PSB2-bRIL-Etrumadenant structure. Of note: The structures of the Imaradenant- and the Vipadenant-complex have been obtained with the A_2A_-StaR2 construct that among other mutations contains a T88^3.36^A point mutation. The structure of the ZM241385-complex showed two different conformations for T88^3.36^. **b** Chemical structures of the depicted antagonists. The dotted blue rectangles highlight structural moieties that form the key hydrogen bonding anchor to N253^6.55^.

Utilizing the optimized construct A_2A_-PSB2-bRIL, we obtained the crystal structure of the A_2A_AR in complex with Etrumadenant at 2.1 Å resolution (see Table 1 for detailed data collection and refinement statistics). Etrumadenant was well resolved within the orthosteric ligand binding pocket (Fig. 2a, b). Its scaffold shows unique interactions within the A_2A_AR’s orthosteric binding pocket. Importantly, the cyano group forms a direct hydrogen bond to T88^3.36^ (N-O distance 2.8 Å) (Fig. 2a, b) representing an interaction that has so far not been observed in A_2A_AR co-crystal structures with various antagonists. T88^3.36^ is conserved within the AR family and was shown to be directly involved in A_2A_AR agonist binding (illustrated for 5’-*N*-ethylcarboxamideadenosine (NECA) in Fig. 2c)^20^. It undergoes significant conformational changes during receptor activation^25^. The interaction of Etrumadenant with T88^3.36^ by direct hydrogen bonding stabilizes the A_2A_AR in its inactive state. Notably, the A_2A_-StaR2-bRIL construct that is extensively used to determine inactive state A_2A_AR crystal structures^16^ harbors a T88^3.36^A mutation (see Fig. 3a), that can be expected to affect the affinity of Etrumadenant and possibly other antagonists. In fact, the affinity of Etrumadenant is ~47-fold lower for A_2A_-StaR2-bRIL as compared to the wt A_2A_AR (K_i_ values of 39.8 nm compared to 0.85 nm, see Table 2). In contrast, the affinity of Etrumadenant for our optimized crystallization construct A_2A_-PSB2-bRIL remained unaltered (K_i_ 1.12 nm).

**Table 1:** Data collection and refinement statistics.

	A_2A_-PSB2-bRIL-Etrumadenant (PDB ID 8C9W)	A_2A_-StaR2-bRIL-A277S-Etrumadenant (PDB ID 8CIC)
*Data collection*		
Space group	C222_1_	C222_1_
Cell dimensions		
* a*, *b*, *c* (Å)	39.16, 178.23, 139.60	39.36, 179.09, 140.57
α, β, γ (°)	90, 90, 90	90, 90, 90
No. of reflections processed	284,104	391,306
No. of unique reflections	21,413	29,632
Resolution (Å)	38.24–2.11 (2.32–2.1)	46.86–2.10 (2.16–2.10)
Max. resolution aniso. (Å)	2.16 (a^*^), 2.11 (b^*^), 2.50 (c^*^)	not applied
*R*_merge_	0.130 (1.697)	0.172 (2.177)
CC_1/2_	0.999 (0.529)	0.999 (0.499)
*I* / σ*I*	13.1 (1.3)	11.1 (1.2)
Completeness spherical	0.748 (0.159)	1.000 (0.996)
Completeness ellipsoidal	0.8980 (0.4125)	Not applicable
Redundancy	13.3 (11.1)	13.2 (13.4)
*Refinement*		
Resolution (Å)	38.24–2.11 (2.23–2.11)	44.78–2.10 (2.14–2.10)
No. of reflections (test set)	21,405 (994)	55,857 (2840)
*R*_work_	0.1965 (0.3298)	0.1904 (0.3081)
*R*_free_	0.2665 (0.5429)	0.2144 (0.3764)
No. atoms (non-hydrogen)		
A_2A_AR	2369	2349
bRIL	689	705
Ligand	32	32
Lipids, polyethylene glycol (PEG) and waters	284	604
*B*-factors (Å²)		
A_2A_AR	40.5	41.8
bRIL	70.8	76.7
Ligand	29.6	32.2
Lipids, PEG and waters	48.3	64.4
Root-mean-square deviation (RMSD)		
Bond lengths (Å)	0.012	0.003
Bond angles (°)	1.305	0.55

^*^For each structure, data from a single crystal was collected. The statistics for the highest resolution shell are shown in parentheses.

**Table 2:** Binding affinities of Etrumadenant and selected antagonists for the human adenosine receptors and for crystallization constructs^a^.

Compounds	Human A_1_AR	Human A_2A_AR	Human A_2B_AR	Human A_3_AR
	[^3^H]DPCPX (or [^3^H]CCPA)	[^3^H]MSX-2	[^3^H]PSB-603	[^3^H]PSB-11 (or [^125^I]-AB-MECA^b^)
	pK_i_ ± SEM	pK_i_ ± SEM	pK_i_ ± SEM	pK_i_ ± SEM
Etrumadenant	8.12 ± 0.08 (8.15 ± 0.02)	9.07 ± 0.14	8.50 ± 0.06	6.50 ± 0.14
ZM241385	6.65^b^	8.69 ± 0.20	7.53 ± 0.20	(< 5.00^b^)
PSB-603	(<5.00^c^)	<5.00^c^	9.26^c^	<5.00^c^
Preladenant	(6.53^d^)	9.05^d^	<6.00^d^	<6.00^d^

	A_2A_-PSB2-bRIL	A_2A_-StaR2-bRIL		
	[^3^H]MSX-2	[^3^H]MSX-2		
	pK_i_ ± SEM	pK_i_ ± SEM		
Etrumadenant	8.95 ± 0.12	7.40 ± 0.05		

^a^pK_i_ values were determined as means from at least three independent experiments ± standard error of the mean (SEM) performed on CHO cell membranes expressing the respective human wt AR, or on *Sf9* insect cell membranes for the two crystallization constructs A_2A_-PSB2-bRIL and A_2A_-StaR2-bRIL. [^3^H]CCPA and [^125^I]-AB-MECA represent agonist radioligands whereas all other radioligands are antagonists at ARs. ^b^Ongini et al.^55^; ^c^Borrmann et al.^29^; ^d^Burbiel et al.^28^.

Besides the hydrogen bond to T88^3.36^, Etrumadenant shows multiple additional receptor-ligand interactions. The phenyl ring of Etrumadenant is stabilized by π-π interactions to H250^6.52^ (T-shaped) and W246^6.48^ (stacked) (Fig. 2b). Its 2-methyl group comes in contact to V84^3.32^, L85^3.33^ and F168^ECL2^. It is additionally exposed to a water network connecting the ligand to helices II and III (Fig. 2a). The 2-aminopyrimidine core is stabilized by π-π stacking interactions to F168^ECL2^ (Fig. 2a) and forms key anchoring interactions by hydrogen bonding of the *N*3 and the exocyclic NH_2_-group to N253^6.55^ (Fig. 2a, b). Hydrogen bonding interactions of the side-chain of N253 are also observed for other ligands including agonists (Fig. 2c) and antagonists (see blue rectangles in Fig. 3). The hydrogen bond network is extended by a direct interaction of the exocyclic NH_2_-group of Etrumadenant with E169^ECL2^ (Fig. 2b). E169^ECL2^ forms a salt bridge to H264^ECL3^ that is frequently observed in A_2A_AR crystal structures, but was found to be dependent on the structure of the antagonist and the pH value during crystallization^16^.

The triazolyl ring of Etrumadenant (Fig. 2d), connected to the 6-position of the core aminopyrimidine, and bearing a substituted pyridylmethylene residue, forms π-π stacking interactions with F168^ECL2^ and water-mediated hydrogen bonding to H278^7.43^ and to the backbones of A59^2.57^, I80^3.28^ and A81^3.29^ (Fig. 2a). The pyridine ring is located in close proximity to the entrance of the orthosteric ligand binding pocket at the extracellular ends of helices I and II with direct contacts to S67^2.65^ and Y271^7.36^. The 2-hydroxyisopropyl residue that is attached to the pyridine of Etrumadenant shows three ambiguous rotamers. We chose to model the rotamer conformation with the hydroxy group in close proximity to a nearby water molecule thereby forming an intramolecular water-mediated hydrogen bond to the pyrimidine *N*1-nitrogen (Fig. 2a, b).

The sidechain of Y271^7.36^ was observed to be highly flexible when comparing different A_2A_AR co-crystal structures^19,26^. It adapts the hydrophobic pocket to the size of the ligand (as depicted for a selection of ligands in Fig. 3). The relatively large Etrumadenant molecule requires a significant sidechain movement of Y271^7.36^. This sidechain is located much closer to the orthosteric binding pocket in the ZM241385-bound A_2A_AR crystal structure, where it is hydrogen-bonded to the water network around the ligand (Fig. 3)^21^. In that structure, an additional oleic acid molecule occupies the space which Y271^7.36^ adopts in the current Etrumadenant structure, where the hydrophilic head group of the oleate is displaced by the rotation of Y271^7.36^ (also compare structures of Imaradenant^26^ and Vipadenant^27^) (Fig. 3).

**Fig. 4:**
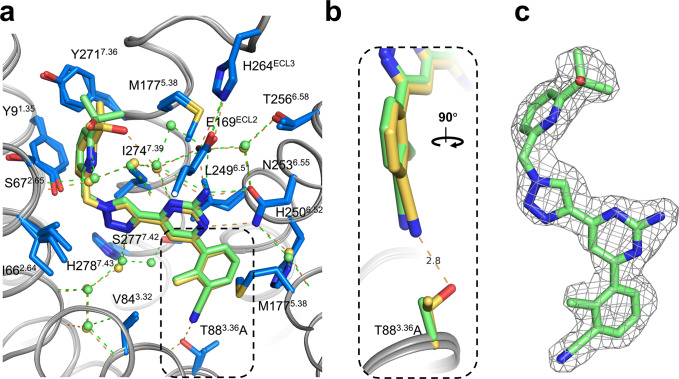
Comparison of the binding pockets of Etrumadenant in the A2A-PSB2-bRIL and A2A-StaR2-bRIL-A277S structures. **a** The binding pose of A_2A_-PSB2-bRIL-Etrumadenant (blue/yellow) is compared to the pose of A_2A_-StaR2-bRIL-A277S-Etrumadenant (blue/green). Green- and yellow-colored dashes represent hydrogen bond interactions. **b** Zoomed panel highlighting the interaction of the cyano group with T88^3.36^ at an N-O distance of 2.8 Å. **c** 2F_o_–F_c_ electron density for Etrumadenant in the A_2A_-StaR2-bRIL-A277S structure, shown as gray mesh and contoured at 1 σ.

Next, we additionally obtained the crystal structure of Etrumadenant in complex with a modified A_2A_-StaR2-bRIL receptor construct in which the S277^7.42^A mutation had been reverted to wt (designated A_2A_-StaR2-bRIL-A277S), but which still harbored the T88^3.36^A mutation in the binding pocket. A co-crystal structure could be obtained at the same high resolution of 2.1 Å (see Table 2 for detailed refinement statistics). Surprisingly, even though a major interaction partner of the ligand was mutated, the binding pockets of A_2A_-PSB2-bRIL-Etrumadenant and A_2A_-StaR2-bRIL-A277S-Etrumadenant are largely similar with only subtle differences (Fig. 4). Notably, the cyano group of Etrumadenant in the A_2A_-PSB2-bRIL structure is slightly tilted, relative to the plane of the phenyl ring, towards the hydroxy group of T88^3.36^ and deviates from the ideal planar orientation by ~8° (Fig. 4b). The same cyano moiety is planar in the T88^3.36^A mutated structure, but is unable to form the same hydrogen bond interaction due to the mutation. Another difference between both structures can be identified in the rotamers of the 2-hydroxyisopropyl residue and the adjacent sidechain of Y271^7.36^ (Fig. 4a) which confirms the initially observed flexibility of these moieties.

### Pharmacological characterization of Etrumadenant

**Fig. 5:**
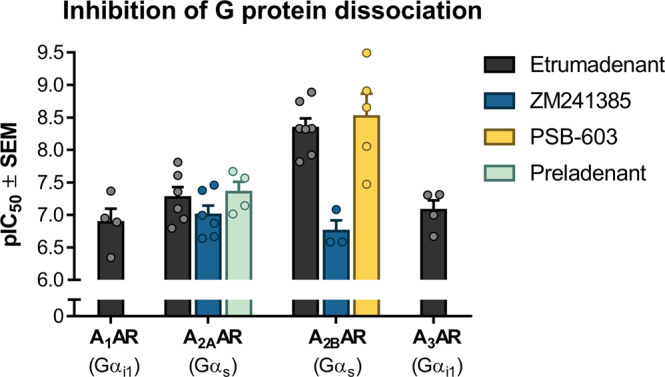
Inhibition of NECA-induced G protein dissociation by adenosine receptor antagonists. pIC_50_ values were determined as means from at least three independent experiments ± SEM using a BRET G protein dissociation assay^32^. Human embryonic kidney (HEK) cells were transfected with the respective AR and Gα-RLuc8, Gβ_3_, and Gγ_9_-GFP2 subunits. In the case of A_1_- and A_3_ARs, Gα_i1_ was used whereas Gα_s_ (short isoform GNAS-2) was used for the A_2A_- and the A_2B_ARs. The receptors were activated by NECA at its EC_80_ for each receptor subtype (A_1_AR 20 nm, A_2A_AR 1 µm, A_2B_AR 5 µm, A_3_AR 20 nm), and concentration-dependent inhibition of the signal by Etrumadenant (or standard antagonists) was observed. EC_80_ values depend on receptor expression^56^ and probably also on G protein expression levels.

In the original patent describing Etrumadenant, affinity ranges were reported, but no specific K_i_ or half-maximal inhibitory concentration (IC_50_) values were provided^14^. In order to complement the pharmacological characterization of Etrumadenant, we determined its affinities for all human AR subtypes as well as for the crystallization constructs by radioligand binding assays (Table 2). To this end, we employed membrane preparations of Chinese hamster ovary (CHO) cells or *Spodoptera frugiperda* (*Sf9*) insect cells recombinantly expressing the respective AR subtype, or crystallization construct, respectively. Additionally, we investigated the inhibitory effects of Etrumadenant in G protein dissociation assays (Fig. 5).

In addition to its high affinity for the A_2A_- and A_2B_AR subtypes confirmed in the present study (K_i_ values: A_2A_, 0.851 nm; A_2B_, 3.16 nm), we found that Etrumadenant also exhibits high affinity for the A_1_AR (K_i_ value: 7.59 nm versus the antagonist radioligand [^3^H]DPCPX, and 7.08 nm versus the agonist radioligand [^3^H]CCPA) (Table 2). Thus, the compound showed only about ninefold selectivity comparing A_2A_- with A_1_AR affinity, and only 2-fold selectivity for the A_2B_- versus A_1_AR subtype. In contrast, we approved that Etrumadenant exhibits high selectivity versus the A_3_AR (>100-fold), as determined in radioligand binding studies. For comparison, we determined the affinities of standard AR antagonists using the same assays (Table 2). While ZM241385 showed a moderate preference for the A_2A_AR (K_i_ values: A_2A_, 2.04 nm; A_2B_, 29.5 nm; 12-fold difference), the A_2A_AR antagonist Preladenant displayed similarly high A_2A_ affinity as Etrumadenant (K_i_: A_2A_, 0.884 nm^28^) but showed high A_2A_-selectivity. The A_2B_AR antagonist PSB-603 was somewhat more potent than Etrumadenant (K_i_: A_2B_, 0.553 nm^29^) showing high selectivity for the A_2B_AR subtype.

Subsequently, functional assays were performed to determine concentration-dependent antagonistic effects of Etrumadenant on receptor activation. To this end, we performed bioluminescence resonance energy transfer (BRET) based G protein dissociation assays employing *Renilla* Luciferase 8 (RLuc8) fused to Gα subunits and green fluorescent protein (GFP) attached to the Gγ subunit^30–32^. AR activation was induced with the non-selective agonist NECA at a concentration where it shows 80% of its maximal effect (EC_80_). The preferentially G_i_ protein-coupled A_1_- and A_3_AR subtypes were co-expressed with Gα_i1_-RLuc8, Gβ_3_, and Gγ_9_-GFP proteins, whereas the Gs protein-coupled A_2A_- and A_2B_AR subtypes were co-expressed with Gα_s_-RLuc8, Gβ_3_, and Gγ_9_-GFP proteins. Etrumadenant was able to block the activation of all four AR subtypes in a concentration-dependent manner. The antagonist was found to be most potent at the A_2A_AR followed by the A_2B_AR, but also showed significant antagonistic activity at the other AR subtypes, A_1_ and A_3_ (see Fig. 5). Blockade of the G_i_ protein-coupled A_1_- and A_3_ARs will lead to an increase in intracellular cyclic adenosine monophosphate (cAMP) levels thereby counteracting the effects of antagonists at the G_s_ protein-coupled A_2A_- and A_2B_ARs^33^. For this reason, A_1_- and A_3_ARs can be regarded as anti-targets in the development of AR antagonists for cancer therapy, and the lack of selectivity may contribute to side-effects^3^.

For comparison, we additionally investigated the prototypical non-selective A_2A_/A_2B_AR antagonist ZM241385, the A_2A_-selective antagonist Preladenant, and the A_2B_-selective antagonist PSB-603. Preladenant inhibited the A_2A_AR with similar potency as Etrumadenant in this assay (IC_50_ values 85.1 nm, 53.7 nm), whereas the potency of ZM241385 (IC_50_: 178 nm) was lower than that of Etrumadenant (IC_50_: 4.57 nm) at the A_2B_AR, but similar at the A_2A_AR (IC_50_ values: 100 nm; 53.7 nm). PSB-603 showed similarly high potency at the A_2B_AR as Etrumadenant (IC_50_ values: 3.02 nm; 4.57 nm). It should be kept in mind that the employed functional G protein activation assays require overexpression of receptors and G proteins^32^. Nevertheless, these data confirm that Etrumadenant is a potent antagonist of A_2A_- and A_2B_ARs, but its selectivity versus the G_i_ protein-coupled ARs is low.

**Fig. 6:**
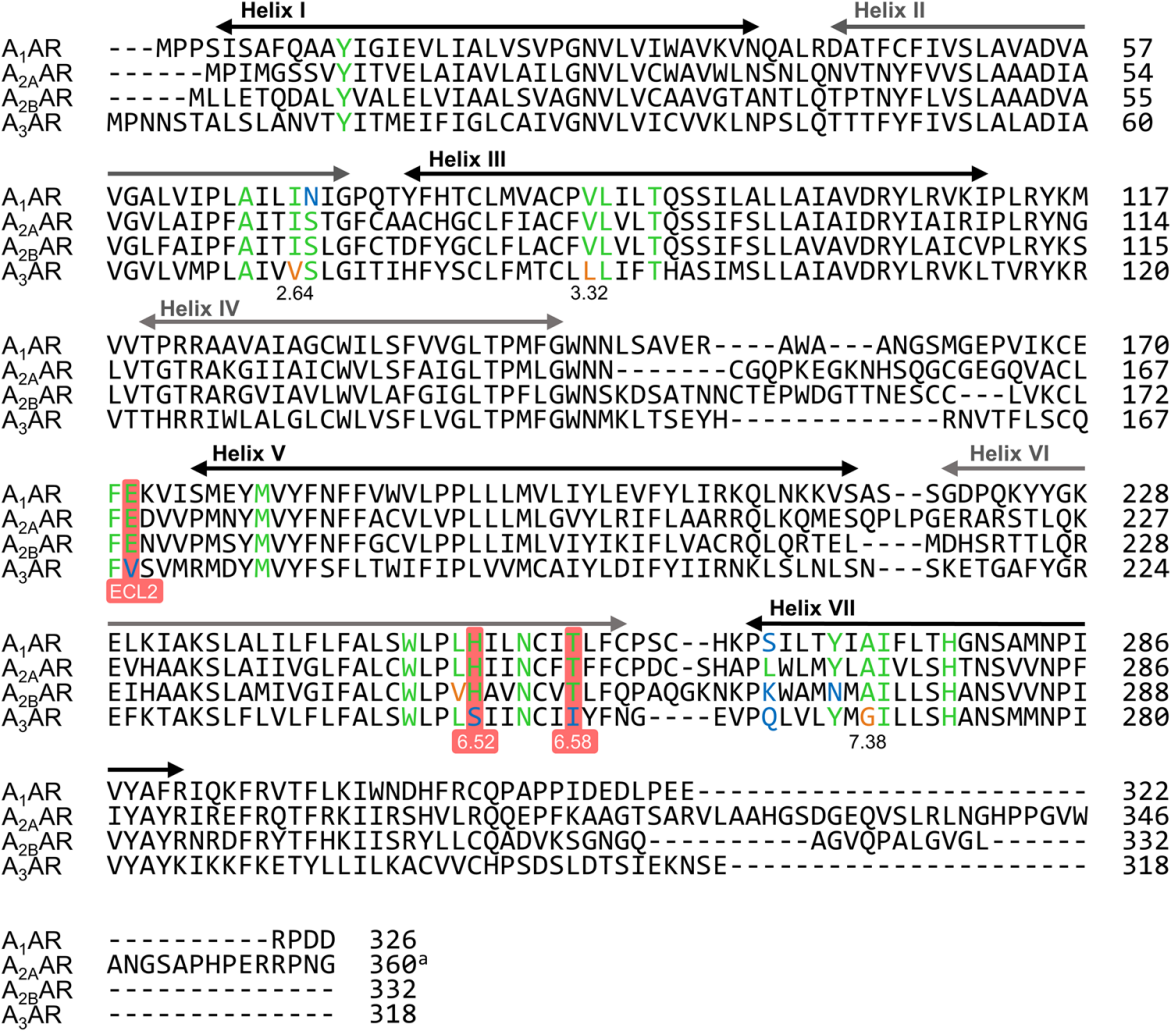
Adenosine receptor sequence alignment. Colored residues indicate amino acids with direct contacts to Etrumadenant or interactions via one structural water molecule as observed in the A_2A_-PSB2-bRIL-Etrumadenant structure. Green colored residues are conserved whereas blue colored residues highlight significant subtype differences. Orange residues indicate exchanges for amino acids with similar properties. Sequences were aligned with Clustal Omega. ^a^The long C-terminal tail of the A_2A_AR (residues 361–412) was omitted from the alignment.

To explain this observation, we performed a sequence alignment of all AR subtypes and analyzed the conservation of amino acids that interact with Etrumadenant as observed in the A_2A_AR co-crystal structures (Fig. 6). In fact, these amino acid residues are largely conserved in the A_1_-, A_2A_-, and A_2B_AR subtypes, which is consistent with the high affinity of Etrumadenant for all three subtypes. The orthosteric binding pockets of the A_2A_- and the A_2B_AR differ only by one homologous amino acid exchange (L249^6.51^ in the A_2A_AR, V250^6.51^ in the A_2B_AR). The recently determined cryogenic electron microscopy structures of the A_2B_AR in the active state confirmed a similar binding mode of the agonists adenosine and NECA in both receptor subtypes^34,35^. The extracellular ends of the A_2B_AR are less conserved, and among the residues that are in contact with Etrumadenant in the A_2A_AR two major differences can be observed: L267^7.32^ and Y271^7.36^ of the A_2A_AR are exchanged for K269^7.32^ and N273^7.36^ present in the A_2B_AR. Therefore, we hypothesize that Etrumadenant’s aminopyridine core exhibits a comparable binding mode in the A_2B_AR as in the A_2A_AR, whereas the substituted pyridylmethylene residue, that extends towards the extracellular space and is relatively flexible, may show differences in binding at both A_2_AR subtypes.

One notable difference between A_1_- and A_2A_ARs is S67^2.65^ of the A_2A_AR that is exchanged for N70^2.65^ in the A_1_AR. S67^2.65^ forms direct contacts with the pyridine core of Etrumadenant at the extracellular ends of the ligand binding pocket. The overall large conservation between the A_1_- and A_2A_AR ligand binding pockets substantiates our observation that Etrumadenant exhibits significant A_1_AR affinity. However, the exchange of S67^2.65^ to N70^2.65^ in the A_1_AR might affect the binding of Etrumadenant and explain the slightly reduced affinity of Etrumadenant for the A_1_AR.

The A_3_AR, on the other hand, shows significant differences being the least conserved AR subtype regarding Etrumadenant’s binding pocket residues. Three hydrophobic amino acid residues that form direct Etrumadenant contacts in the A_2A_AR (I66^2.64^, V84^3.32^, and A273^7.38^) are exchanged in the A_3_AR for different hydrophobic amino acids or glycine (V72^2.64^, L90^3.32^, and G267^7.38^). Moreover, we observed direct interactions of Etrumadenant to the side chains of E169^ECL2^ and H250^6.52^ as well as a water-bridged hydrogen bond to T256^6.58^. These residues are conserved among the A_1_-, A_2A_-, and A_2B_ARs, whereas the A_3_AR contains V169^ELC2^, S247^6.52^, and I253^6.58^ in the analogous positions (Fig. 4, red boxes). Variation of these interacting residues in the A_3_AR provides an explanation for the decreased affinity of Etrumadenant for the A_3_AR.

## Conclusions

The adenosine receptor antagonist Etrumadenant represents a promising clinical candidate for the treatment of cancer, with high affinity for A_2A_ and A_2B_ARs. Both AR subtypes represent purinergic immune checkpoints inhibiting the immune system and showing ancillary direct cancer proliferation-enhancing and pro-angiogenic effects^7,33^. Blockade of A_2A_- and A_2B_ARs consequently is expected to exert anti-cancer activity. We show that the affinity of Etrumadenant for the A_1_AR, which has been discussed as an anti-target in cancer therapy, is similarly high as for the A_2A_- and A_2B_AR, with a K_i_ value in the single-digit nanomolar range. The first A_2A_AR co-crystal structures of Etrumadenant in complex with the A_2A_AR provide insights into the A_2A_AR ligand binding pocket. They revealed that Etrumadenant stabilizes the inactive state of the A_2A_AR by hydrogen bond interaction to T88^3.36^ through its cyano group. A direct hydrogen bond to T88^3.36^ has thus far not been observed in antagonist-bound A_2A_AR crystal structures. Importantly, the A_2A_-StaR2 construct that has been used for the vast majority of A_2A_AR co-crystal structures^16^ contains two mutations inside the ligand binding pocket (T88^3.36^A and S277^7.42^A) preventing the discovery of this hydrogen bond. In fact, out of the 24 different A_2A_AR antagonists for which co-crystal structures have been solved to date (see Supplementary Table 1) only five have been determined with A_2A_AR constructs harboring the native T88^3.36^ (ZM241384^21^, “cmpd-1”^36^, PSB-2113^16^, PSB-2115^16^, and most recently KW-6356^37^). Nevertheless, the structure of a modified A_2A_-StaR2-bRIL construct (that has the S277^7.42^A mutation reverted to the wt residue) in complex with Etrumadenant revealed nearly identical binding poses of Etrumadenant, despite the T88^3.36^A mutation. However, we show that the affinity of Etrumadenant for the A_2A_-StaR2-bRIL crystallization construct is reduced by 47-fold compared to the wt A_2A_AR (K_i_ 39.8 nm vs. 0.851 nm) whereas the affinity to the employed optimized crystallization construct A_2A_-PSB2-bRIL is unaltered (K_i_ 1.12 nm). The discovered interaction with T88^3.36^ will be relevant for the design and optimization of future A_2A_AR antagonists as well as for dual A_2A_/A_2B_AR antagonists and pan-AR antagonists, in particular, since T^3.36^ is conserved among the AR subtypes, not only in humans, but also in rats^38^. Its conservation likely also contributes to Etrumadenant’s high affinity for the A_1_AR. The A_3_AR contains several non-conserved residues that are involved in A_2A_AR binding, which could explain the selectivity of Etrumadenant versus the A_3_AR subtype as observed in radioligand binding experiments (more than two orders of magnitude). The present X-ray structure will serve as a basis for the future design of tailored AR antagonists, which have great potential for the treatment of cancer as well as neurodegenerative diseases.

## Methods

### Expression, purification and crystallization of the A_2A_-PSB2-bRIL-Etrumadenant complex

The crystallization construct A_2A_-PSB2-bRIL was cloned using site-directed mutagenesis in order to add the glycosylation mutation N154^ECL2^A to the previously reported crystallization construct A_2A_-PSB1-bRIL^16^. The N154^ECL2^Q and N154^ECL2^D mutations were cloned analogously. A_2A_-PSB2-bRIL was expressed and purified in analogy to the procedure described for A_2A_-PSB1-bRIL^16^. Briefly, A_2A_-PSB2-bRIL was expressed in *Sf9* insect cells using GP64-pFastBac1 as baculoviral expression vector. Cells were disrupted by osmotic shock and membranes were repeatedly washed using a washing buffer that contained a high amount of NaCl. The resuspended cell membranes were subsequently incubated with 50 µm Etrumadenant (obtained from MedChemExpress, cat. #HY-129393) and 2 mg mL^−1^ iodoacetamide for 1 h prior to solubilization. A_2A_-PSB2-bRIL was solubilized and purified from *Sf9* membranes similarly as described for A_2A_-PSB1-bRIL^16^. Etrumadenant was added to wash buffer I and to wash buffer II at 50 µm and 25 µm concentration, respectively. The protein was eluted with four column volumes of elution buffer containing 25 µm Etrumadenant, 25 mm HEPES pH 7.5, 800 mm NaCl, 10% (v/v) glycerol, 220 mm imidazole, 0.025% (w/v) dodecyl-β-d-maltoside (DDM), and 0.005% (w/v) cholesteryl hemisuccinate (CHS).

The A_2A_-PSB2-bRIL-Etrumadenant complex was concentrated to a volume of 20–30 µL using 100 kDa cut-off Vivaspin concentrators (Sartorius), and immediately used for crystallization experiments. The complex was reconstituted into lipidic cubic phase using the two-syringe method^39^ by mixing the protein with a molten lipid mixture [90% (w/w) 1-oleoyl-*rac*-glycerol (Sigma), 10% (w/w) cholesterol (Sigma)] in a 2 to 3 ratio. Crystallization experiments were performed using an automatic crystallization robot (Formulatrix NT8) by overlaying 50 nL of mesophase with 800 nL of precipitant solution. The A_2A_-PSB2-bRIL-Etrumadenant complex crystallized in 30% (w/v) PEG400, 7% (w/v) Tacsimate pH 7.0 (Hampton Research, cat. #HR2-755)^40^, 100 mm HEPES-Na pH 7.4, 1.8% (w/v) 2,5-hexandiol (Molecular Dimensions, cat. #MD2-100-226). Crystals were harvested with micromounts (MiTeGen) and flash-frozen in liquid nitrogen without further cryo-protection.

### X-ray diffraction data collection and structure elucidation of the A_2A_-PSB2-bRIL-Etrumadenant structure

X-ray data collection was carried out at 100 K on EMBL beamline P14 of the DESY synchrotron (Hamburg, Germany). The x-ray wavelength used for the experiment was 0.97625 Å. An Eiger2 16 M detector was placed at a distance of 340 mm behind the crystal, which was rotated for 360° while diffraction images were recorded at 0.15° steps with exposure for 0.01 s. All datasets were indexed, integrated, scaled, and converted to structure factor amplitudes using ISPyB software: autoPROC^41^, XDS^42^, CCP4^43^, POINTLESS^44^, AIMLESS^45^, STARANISO^46^. Crystallographic statistics are presented in Table 1. PDB ID 5IU4^47^ was used as the starting model for refinement with phenix^48^. Coot^49^ was used for model building. The stereochemical restraints for the ligand were generated with the GRADE web server^50^. The Ramachandran plot statistics were determined to 97.65% (favored), 2.09% (allowed), and 0.26% (disallowed).

### Expression, purification and crystallization of the A_2A_-StaR2-bRIL-A277S-Etrumadenant complex

A_2A_ construct (A_2A_-StaR2-bRIL-A277S) containing the same thermostabilizing and deglycosylation site mutations as PDB ID 5IU4^27^ (except for the S277^7.42^A mutation, which is reverted to wt) was codon-optimized and cloned between the BamHI and HindIII sites of pFastBac1 (Trenzyme). The bacmid was generated by transforming this plasmid into *E. coli* strain DH10EMBacY (MultiBac, Geneva Biotech). Isolated bacmid DNA was transfected into *Sf9* insect cell using Cellfectin (Invitrogen) to generate baculovirus. For large-scale expression, High Five insect cells growing at 27 °C in Sf900 II medium (Invitrogen) at 2.5 ∙ 10^6^ ∙ mL^−1^ were infected with P2 baculovirus and harvested at 48 h post infection. Cells were harvested by centrifugation and the pellet was stored at −80 °C. Cells were thawed at room temperature and resuspended in 40 mm Tris-HCl pH 7.6, 1 mm ethylenediaminetetraacetic acid (EDTA), and cOmplete EDTA-free protease inhibitors tablets (Roche). Membranes were fractionated by passing the cell once using microfluidizer (Microfluidics) operated at 8000 psi. Membranes were centrifuged at 42000 rpm using a Ti45 rotor (Beckman) and washed once with 40 mm Tris-HCl pH 7.6, 1 M NaCl, and cOmplete EDTA-free protease inhibitors tablets. Membranes were centrifuged again and resuspended in 40 mm Tris−HCl pH 7.6, cOmplete EDTA-free protease inhibitor cocktail tablets and frozen at −80 °C. Unless otherwise stated, all purification procedures were carried out at 4 °C. Membranes were solubilized with 1.5% (w/v) decylmaltoside (DM) and 0.1% (w/v) CHS for 1 h. Insoluble fractions were pelleted by centrifugation at 42,000 rpm using a Ti45 rotor (Beckman) for 30 min. A_2A_-StaR2-bRIL-A277S was purified by loading the supernatant (supplemented with 8 mm imidazole) into a 5 mL cartridge containing Ni-NTA super flow resin (Qiagen) at 2 mL min^−1^ using an ÄKTA pure system (Cytiva). The resin was first washed with 25 mL of 40 mm Tris-HCl pH 7.5, 400 mm NaCl, 0.15% (w/v) DM, 0.002% (w/v) CHS, and 8 mm imidazole and then washed once more with 25 mL of 40 mm Tris-HCl pH 7.5, 400 mm NaCl, 0.% (w/v) DM, 0.002% (w/v) CHS, and 72 mm imidazole. The protein was eluted in the same buffer containing 272 mm imidazole, concentrated using Vivaspin turbo 15 mL with a molecular weight cut-off of 50 kDa (Sartorius) and loaded onto a Superdex 200 10/300 GL increase column equilibrated in 40 mm Tris-HCl pH 7.4, 200 mm NaCl, 0.15% (w/v) DM, and 0.002% (w/v) CHS. Fractions containing A_2A_-StaR2-bRIL-A277S were pooled, concentrated to 22.5 mg ml^−1^ aliquots and frozen at −80 °C. Protein purity and homogeneity were controlled by SDS-PAGE and fluorescence size exclusion chromatography (FSEC).

For crystallization, frozen A_2A_-StaR2-bRIL-A277S aliquots were thawed on ice and centrifuged at 18,213×*g* for 10 min. Lipidic cubic phase (LCP) crystallization was performed by mixing the protein into monoolein (containing 10% (w/w) cholesterol) at 2:3 (w/w) ratio. The resulting LCP was dispensed using the mosquito LCP (SPT Labtech) using a bolus/precipitant solution ratio of 40:800 nL. Crystals were obtained using precipitant solution containing 0.1 M sodium citrate pH 5.0, 50 mm sodium thiocyanate, 3% (v/v) 2-methyl-2,4-pentanediol (MPD), 21–32% (w/v) PEG400, and 2 mm theophylline. Crystals appeared overnight and grew to full size (50–60 µm in the longest dimension) over a week. To prepare the A_2A_-StaR2-bRIL-A277S-Etrumadenant complex, crystals were soaked overnight in the same precipitant solution by replacing theophylline with 1 mm Etrumadenant^27^. After soaking overnight, crystals were harvested with MicroLoops LD (mitogen) and frozen in liquid nitrogen.

### X-ray diffraction data collection and structure elucidation of the A_2A_-StaR2-bRIL-A277S-Etrumadenant structure

Diffraction data were collected at the Swiss Light Source (SLS) beamline PXII. Crystal was exposed to a 25 × 13 µm X-ray beam (wavelength 0.99997 Å) at 25% transmission. A total of 180° of rotational data were collected using 0.1° oscillation and 0.05 s exposure per image. Dataset was processed and scaled to 2.1 Å using *XDS*^42^ (built 20161205), then merged and converted to mtz file format with *AIMLESS*^45^ (version 0.7.3 from *CCP4*^43^ distribution 7.0.066). The structure was solved by molecular replacement with *PHASER*^51^ (version 2.8.2 from *CCP4* distribution 7.0.066), using PDB ID 5IU4^27^ as the search model. The model was rebuilt and refined using COOT^49^ and *PHENIX*^52^ (version 1.14) using TLS and optimizing 8CIC and ADP weight. After structure refinement, the model was validated using *MolProbity*^53^ (from PHENIX version 1.14). The Ramachandran plot statistics were determined to 99.48% (favored), 0.52% (allowed), and 0% (disallowed).

### Radioligand binding studies

Radioligand binding assays were performed on CHO cell membranes or *Sf9* insect cell membranes expressing the respective human wt adenosine receptor or A_2A_AR crystallization constructs (A_2A_-PSB2-bRIL or A_2A_-StaR2-bRIL)^16,38^. The agonist [^3^H]CCPA or the antagonist [^3^H]DPCPX were employed as radioligands for the A_1_AR (at 1 nm and 0.4 nm final concentration, respectively), the antagonist [^3^H]MSX-2 was used for the A_2A_AR (at 1 nm final concentration), the antagonist [^3^H]PSB-603 for the A_2B_AR (at 0.3 nm final concentration) and the antagonist [^3^H]PSB-11 for the A_3_AR (at 0.5 nm final concentration). All assays were performed in 50 mm Tris buffer (pH 7.4 at room temperature) at a final volume of 400 µL (A_1_-, A_2A_-, and A_3_ARs) or 1000 µL (A_2B_AR). Test compounds were dissolved in dimethyl sulfoxide and incubated at room temperature with the respective membranes and radioligand for 90 min (A_1_AR, [^3^H]CCPA), 60 min (A_1_AR, [^3^H]DPCPX), 30 min (A_2A_AR), 75 min (A_2B_AR) and 45 min (A_3_AR). The final dimethyl sulfoxide concentration was 1%. Then, the mixture was filtered through GF/B glass fiber filters using a cell harvester (Brandel). For the A_2A_AR assay, filters were pre-soaked in an aqueous solution of 0.3% (w/v) of polyethyleneimine for at least 30 min to reduce non-specific binding. Filters were then washed three times with 2 mL ice-cold Tris buffer (50 mm, pH 7.4 at room temperature). Filters containing the A_2B_AR were washed with the same ice-cold Tris buffer but with the addition of 0.1% BSA. The remaining radioactivity was quantified after incubation for 9 h with scintillation cocktail (Beckmann Coulter) using a scintillation counter (Tricarb 2700TR).

### G protein dissociation assays

TRUPATH BRET² assays^32^ were performed as previously described^54^ (TRUPATH was a gift from Bryan Roth (Addgene kit #1000000163)). In case of Gα_i/o_-coupled adenosine receptors (A_1_ and A_3_ARs), a biosensor composed of Gα_i1_-RLuc8, Gβ_3_, and Gγ_9_-GFP2 was used. In case of the Gα_s_-coupled A_2A_- and A_2B_ARs, the biosensor consisted of Gα_s_-RLuc8, Gβ_3_, and Gγ_9_-GFP2. Antagonist solution was incubated with the cells for 20 min before the addition of luciferase substrate solution (50 µm Deep Blue C, Biomol). Agonist solution (NECA) was added 5 min after the addition of the substrate solution at its EC_80_ concentrations (A_1_AR 20 nm, A_2A_AR 1 µm, A_2B_AR 5 µm, A_3_AR 20 nm) and incubated for 5 min until measurement. GFP2 fluorescence (515 nm emission filter) was divided by RLuc8 luminescence (395 nm emission filter) to obtain BRET ratios. Data was normalized to controls (100% activation = NECA without antagonist, 0% activation = no agonist), and IC_50_ values were obtained by a four-parameter sigmoidal curve fit in GraphPad PRISM v8.0 (GraphPad, San Diego, CA). The Gα_s_ biosensor appeared to display a markedly lower expression level than the Gα_i_ biosensor.

### SDS-PAGE analysis

Proteins for SDS-PAGE were expressed and purified from 40 mL of *Sf9* insect cell culture for thermostability assessment^16^. Proteins were analyzed on homemade 10% SDS-PAGE gels casted using *bis*-2-amino-2-(hydroxymethyl)propane-1,3-diol (*bis*-Tris) buffer. Protein samples were prepared using NuPAGE loading dye (ThermoFisher, cat. #NP0007) supplemented with a final concentration of 50 mm dithiothreitol (DTT). Samples were incubated for 30 min at 37 °C prior to SDS-PAGE analysis using 3-(*N*-morpholino)propanesulfonic acid (MOPS) running buffer without addition of sodium hydrogen sulfite. SDS-PAGE gels were stained with Coomassie brilliant blue R-250 and destained using hot water. In order to investigate the effect of Tunicamycin on A_2A_AR glycosylation, the respective insect cell culture was treated with 1 µg mL^−1^ of Tunicamycin (CaymanChemical, cat. #11445) during infection. PNGase F (New England Biolabs, cat. #P0704S) was used to cleave the glycosylation in the purified protein prior to SDS-PAGE analysis using 375 units in a total reaction volume of 22.5 µL followed by incubation at 16 °C for 16 h.

## Supplementary information

Peer Review File

Supplemental Information

## Data Availability

The datasets generated during and/or analyzed during the current study are available from the corresponding author on reasonable request. The coordinates and structure factors for the obtained crystal structures have been deposited to the Protein Data Bank (https://www.rcsb.org/) under accession IDs 8C9W (A_2A_-PSB2-bRIL-Etrumadenant) and 8CIC (A_2A_-StaR2-bRIL-A277A).
