# Supplementary material for: Crystal structure of adenosine A2A receptor in complex with clinical candidate Etrumadenant reveals unprecedented antagonist interaction
Source: Commun Chem. 2023 Jun 1;6:106. doi: 10.1038/s42004-023-00894-6 (PMC10235125; doi:10.1038/s42004-023-00894-6)
Supplement: Supplementary file 2 — Supplemental Information [file 42004_2023_894_MOESM2_ESM.pdf]

Supplementary Information

Crystal structure of adenosine A_{2A} receptor in complex with clinical candidate Etrumadenant reveals unprecedented antagonist interaction

Tobias Claff[1]*, Jonathan G. Schlegel[1], Jan H. Voss[1], Victoria J. Vaaßen[1], Renato H. Weiße[2], Robert K. Y. Cheng[3], Sandra Markovic-Mueller[3], Denis Bucher[3], Norbert Sträter[2], and Christa E. Müller[1]*

[1] *PharmaCenter Bonn & Pharmaceutical Institute, Department of Pharmaceutical & Medicinal Chemistry, University of Bonn, An der Immenburg 4, 53113, Bonn, Germany.*

[2] *Institute of Bioanalytical Chemistry, Center for Biotechnology and Biomedicine, University of Leipzig, Deutscher Platz 5, 04103, Leipzig, Germany.*

[3] *leadXpro AG, PARK InnovAARE, 5234 Villigen, Switzerland.*

*Correspondence: christa.mueller@uni-bonn.de; tobias.claff@uni-bonn.de

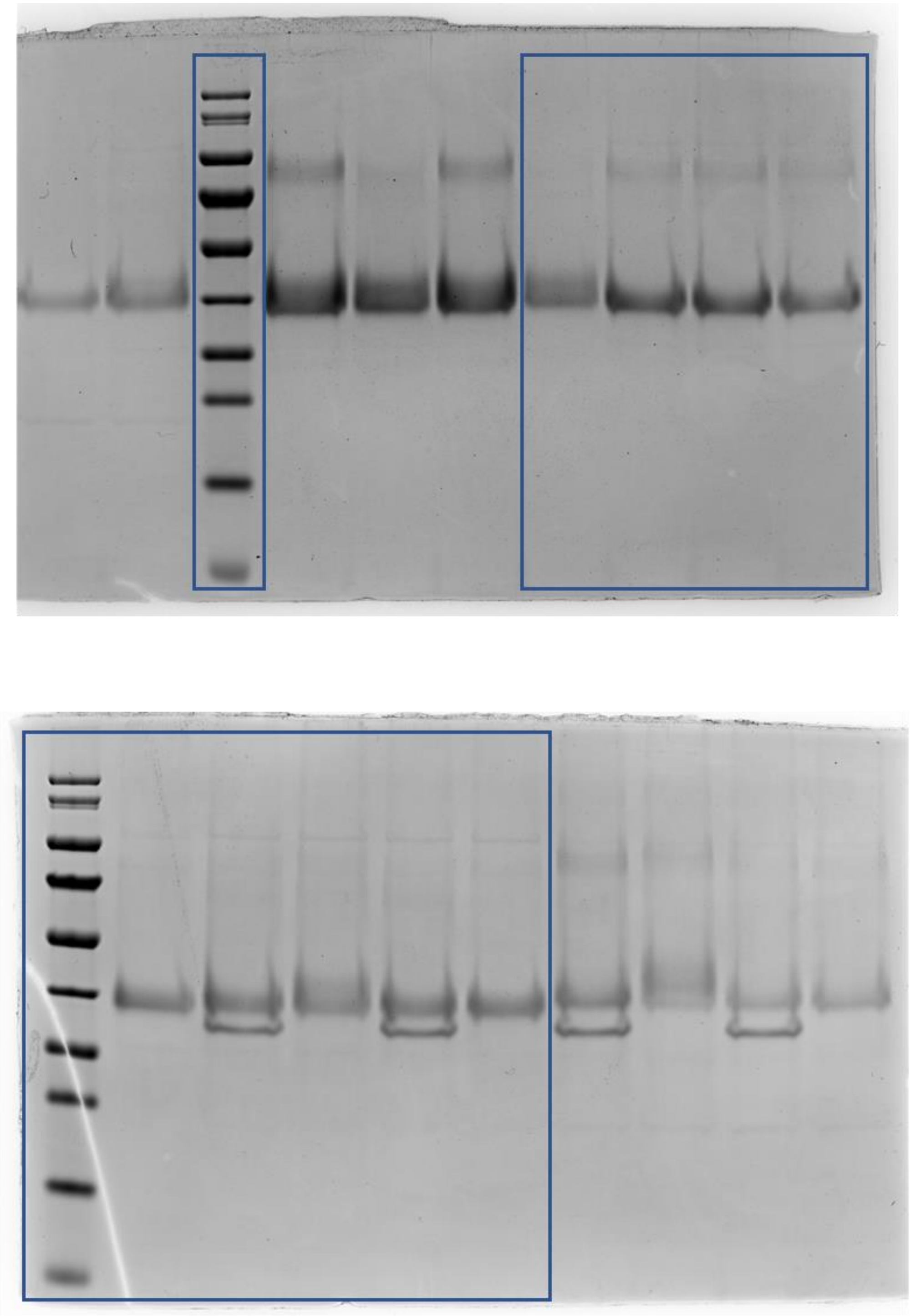

Supplementary Figure 1. SDS-PAGE analysis of the $A_{2A}AR$ glycosylation state (uncropped SDS-PAGE images). The cropped sections used in Figure 1 of this manuscript are indicated by blue rectangles. It should be noted that the other protein samples present in the SDS-PAGE images are not related to this study.

Supplementary Table 1. List of all published A_{2A}AR crystal structures in complex with A_{2A}AR antagonists in the Protein Data Bank (PDB) as of April 15, 2023.[a]

PDB identifier	Ligand	A_{2A}AR construct[b]	Resolution (Å)	H264-E169 ionic lock	pH during crystallization	References
3EML	ZM241385	A_{2A}-ΔC-T4L	2.60	intact	5.5 – 6.5	1
4EIY 5JTB 6WQA<br>5K2A 5VRA 6LPJ<br>5K2B 6AQF 6LPK<br>5K2C 6MH8 6LPL<br>5K2D 6JZH 7RM5<br>5UVI 6PS7 8FYN	ZM241385	A_{2A}-ΔC-bRIL (+ N154Q in 6AQF)	1.80 – 4.20	intact	4.8 – 5.0	2–14
3PWH	ZM241385	A_{2A}-StaR2	3.30	open	8.0 – 8.75	15
3VGA 3VG9	ZM241385	A_{2A}-ΔC + N154Q	2.70 – 3.10	intact/ open	6.5	16
5IU4 5NM4 6S0L<br>5NLX 5OLG 6S0Q<br>5NM2	ZM241385	A_{2A}-StaR2-bRIL	1.72 – 2.14	intact	5.0 – 5.4	17–19
3REY	Xanthine amine congener (XAC)	A_{2A}-StaR2	3.31	open	8.0 – 8.75	15
3RFM	Caffeine	A_{2A}-StaR2	3.60	open	8.0 – 8.75	15
5MZP	Caffeine	A_{2A}-StaR2-bRIL	2.10	intact	5.0	20
3UZA	4g	A_{2A}-StaR2	3.27	open	8.0 – 8.75	21
3UZC	4e	A_{2A}-StaR2	3.34	open	8.0 – 8.75	21
5OLZ	4e	A_{2A}-StaR2-bRIL	1.90	intact	5.3 – 5.4	19
5OM1	4e	A_{2A}-StaR2-bRIL	2.10	intact	5.3 – 5.4	19
5OM4	4e	A_{2A}-StaR2-bRIL	2.00	intact	5.3 – 5.4	19
5IU7	12c	A_{2A}-StaR2-bRIL	1.90	intact	5.3 – 5.4	17
5IU8	12f	A_{2A}-StaR2-bRIL	2.00	intact	5.5	17
5IUA	12b	A_{2A}-StaR2-bRIL	2.20	intact	5.3 – 5.4	17
5IUB	12x	A_{2A}-StaR2-bRIL	2.10	intact	5.5	17
5MZJ	Theophylline	A_{2A}-StaR2-bRIL	2.00	intact	5.1	20
5N2R	PSB-36	A_{2A}-StaR2-bRIL	2.80	open	5.1	20
5OLH	Vipadenant	A_{2A}-StaR2-bRIL	2.60	intact	5.3 – 5.4	19
5OLO	Tozadenant	A_{2A}-StaR2-bRIL	3.10	open	5.3 – 5.4	19
5OLV	LUAA47070	A_{2A}-StaR2-bRIL	2.00	intact	5.3 – 5.4	19
5UIG	Cmpd-1	A_{2A}-ΔC-bRIL (modified N- and C- terminus	3.50	intact	6.5	22
6GT3	Imaradenant	A_{2A}-StaR2-bRIL	2.00	intact	5.3 – 5.4	23
6ZDR	Chromone 4d	A_{2A}-StaR2-bRIL	1.92	intact	4.7 – 5.4	24
6ZDV	Chromone 5d	A_{2A}-StaR2-bRIL	2.13	intact	4.7 – 5.4	24

PDB identifier	Ligand	A_{2A}AR construct[b]	Resolution (Å)	H264-E169 ionic lock	pH during crystallization	References
7PX4	PSB-2113	A_{2A}-PSB1-bRIL	2.25	intact	5.2	[25]
7PYR	PSB-2115	A_{2A}-PSB1-bRIL	2.60	intact	5.2	[25]
8CU6	LJ-4517	A_{2A}-StaR2-bRIL-A277S	2.80	intact	5.3	[26]
8CU7	LJ-4517	A_{2A}-StaR2-bRIL	2.05	intact	5.3	[26]
8DU3	21a	A_{2A}-StaR2-bRIL	2.50	intact	4.5	[27]
8GNE	KW-6356	A_{2A}-ΔC-bRIL + N154Q	2.30	open	5.0	[28]
8GNG	Istradefylline	A_{2A}-Rant21-bRIL	3.20	intact	7.0	[28]
8C9W	Etrumadenant	A_{2A}-PSB2-bRIL	2.11	intact	7.4	this paper
8CIC	Etrumadenant	A_{2A}-StaR2-bRIL-A277S	2.10	intact	5.0	this paper

[a]Table was complemented based on Claff et al.[25]

[b]The A_{2A}AR constructs contain the following point mutations:

A_{2A}-ΔC-T4L:	no point mutations
A_{2A}-ΔC-bRIL:	no point mutations
A_{2A}-StaR2-bRIL:	$A54^{2.42}L$, $\mathbf{T88^{3.36}A}$, $R107^{3.55}A$, $K122^{4.43}A$, $N154^{ECL2}A$, $L202^{5.63}A$, $L235^{6.37}A$, $V239^{6.41}A$, $S277^{7.42}A$
A_{2A}-StaR2-bRIL-A277S:	$A54^{2.42}L$, $\mathbf{T88^{3.36}A}$, $R107^{3.55}A$, $K122^{4.43}A$, $N154^{ECL2}A$, $L202^{5.63}A$, $L235^{6.37}A$, $V239^{6.41}A$
A_{2A}-Rant21-bRIL:	$A54^{2.42}L$, $\mathbf{T88^{3.36}A}$, $K122^{4.43}A$, $V239^{6.41}A$)
A_{2A}-PSB1-bRIL:	$S91^{3.39}K$
A_{2A}-PSB2-bRIL:	$S91^{3.39}K$, $N154^{ECL2}A$

Supplementary References

1. Jaakola, V.-P. *et al.* The 2.6 angstrom crystal structure of a human A_{2A} adenosine receptor bound to an antagonist. *Science* **322,** 1211–1217 (2008).
2. Martynowycz, M. W. *et al.* A robust approach for MicroED sample preparation of lipidic cubic phase embedded membrane protein crystals. *Nat. Commun.* **14,** 1086 (2023).
3. Liu, W. *et al.* Structural basis for allosteric regulation of GPCRs by sodium ions. *Science* **337,** 232–236 (2012).
4. Batyuk, A. *et al.* Native phasing of x-ray free-electron laser data for a G protein-coupled receptor. *Sci. Adv.* **2,** e1600292 (2016).
5. Martin-Garcia, J. M. *et al.* Serial millisecond crystallography of membrane and soluble protein microcrystals using synchrotron radiation. *IUCrJ* **4,** 439–454 (2017).
6. Melnikov, I. *et al.* Fast iodide-SAD phasing for high-throughput membrane protein structure determination. *Sci. Adv.* **3,** e1602952 (2017).
7. Broecker, J. *et al.* High-throughput in situ X-ray screening of and data collection from protein crystals at room temperature and under cryogenic conditions. *Nat. Protoc.* **13,** 260–292 (2018).
8. White, K. L. *et al.* Structural connection between activation microswitch and allosteric sodium site in GPCR signaling. *Structure* **26,** 259-269.e5 (2018).
9. Martin-Garcia, J. M. *et al.* High-viscosity injector-based pink-beam serial crystallography of microcrystals at a synchrotron radiation source. *IUCrJ* **6,** 412–425 (2019).
10. Shimazu, Y. *et al.* High-viscosity sample-injection device for serial femtosecond crystallography at atmospheric pressure. *J. Appl. Crystallogr.* **52,** 1280–1288 (2019).
11. Ishchenko, A. *et al.* Toward G protein-coupled receptor structure-based drug design using X-ray lasers. *IUCrJ* **6,** 1106–1119 (2019).
12. Lee, M.-Y. *et al.* Harnessing the power of an X-ray laser for serial crystallography of membrane proteins crystallized in lipidic cubic phase. *IUCrJ* **7,** 976–984 (2020).
13. Ihara, K. *et al.* Isoprenoid-chained lipid EROCOC17+4: a new matrix for membrane protein crystallization and a crystal delivery medium in serial femtosecond crystallography. *Sci. Rep.* **10,** 19305 (2020).

14. Martynowycz, M. W. *et al.* MicroED structure of the human adenosine receptor determined from a single nanocrystal in LCP. *Proc. Natl. Acad. Sci. U. S. A.* **118** (2021).

15. Doré, A. S. *et al.* Structure of the adenosine A_{2A} receptor in complex with ZM241385 and the xanthines XAC and caffeine. *Structure* **19,** 1283–1293 (2011).

16. Hino, T. *et al.* G-protein-coupled receptor inactivation by an allosteric inverse-agonist antibody. *Nature* **482,** 237–240 (2012).

17. Segala, E. *et al.* Controlling the dissociation of ligands from the adenosine A_{2A} receptor through modulation of salt bridge strength. *J. Med. Chem.* **59,** 6470–6479 (2016).

18. Weinert, T. *et al.* Serial millisecond crystallography for routine room-temperature structure determination at synchrotrons. *Nat. Commun.* **8,** 542 (2017).

19. Rucktooa, P. *et al.* Towards high throughput GPCR crystallography: In meso soaking of adenosine A_{2A} receptor crystals. *Sci. Rep.* **8,** 41 (2018).

20. Cheng, R. K. Y. *et al.* Structures of human A_1 and A_{2A} adenosine receptors with xanthines reveal determinants of selectivity. *Structure* **25,** 1275-1285.e4 (2017).

21. Congreve, M. *et al.* Discovery of 1,2,4-triazine derivatives as adenosine A_{2A} antagonists using structure based drug design. *J. Med. Chem.* **55,** 1898–1903 (2012).

22. Sun, B. *et al.* Crystal structure of the adenosine A_{2A} receptor bound to an antagonist reveals a potential allosteric pocket. *Proc. Natl. Acad. Sci. U. S. A.* **114,** 2066–2071 (2017).

23. Borodovsky, A. *et al.* Small molecule AZD4635 inhibitor of $A_{2A}R$ signaling rescues immune cell function including $CD103^+$ dendritic cells enhancing anti-tumor immunity. *J. Immunother. Cancer* **8** (2020).

24. Jespers, W. *et al.* X-ray crystallography and free energy calculations reveal the binding mechanism of A_{2A} adenosine receptor antagonists. *Angew. Chem. Int. Ed.* **59,** 16536–16543 (2020).

25. Claff, T. *et al.* Single stabilizing point mutation enables high-resolution co-crystal Structures of the adenosine A_{2A} receptor with preladenant conjugates. *Angew. Chem. Int. Ed.,* e202115545 (2022).

26. Shiriaeva, A. *et al.* GPCR agonist-to-antagonist conversion: enabling the design of nucleoside functional switches for the A_{2A} adenosine receptor. *J. Med. Chem.* **65,** 11648–11657 (2022).

27. Bolteau, R. *et al.* High ligand efficiency quinazoline compounds as novel A_{2A} adenosine receptor antagonists. *Eur. J. Med. Chem.* **241,** 114620 (2022).

28. Ohno, Y. *et al.* In vitro pharmacological profile of KW-6356, a novel adenosine A_{2A} receptor antagonist/inverse agonist. *Mol. Pharmacol.* (2023).
